# The *Ve*-mediated resistance response of the tomato to *Verticillium dahliae *involves H_2_O_2_, peroxidase and lignins and drives *PAL *gene expression

**DOI:** 10.1186/1471-2229-10-232

**Published:** 2010-10-26

**Authors:** Carmen Gayoso, Federico Pomar, Esther Novo-Uzal, Fuencisla Merino, Óskar Martínez de Ilárduya

**Affiliations:** 1 Departamento de Biología Animal, Biología Vegetal y Ecología, Universidad de La Coruña, 15071 La Coruña, Spain; 2 Instituto de Investigaciones Biomédicas de A Coruña (INIBIC), Complejo Hospitalario Universitario de A Coruña, As Xubias s/n, 15006 La Coruña, Spain; 3 Networking Center of Biomedical Research in Bioengineering, Biomaterials and Nanomedicine (CIBER-BBN), 15006 La Coruña, Spain

## Abstract

**Background:**

*Verticillium dahliae *is a fungal pathogen that infects a wide range of hosts. The only known genes for resistance to *Verticillium *in the Solanaceae are found in the tomato (*Solanum lycopersicum*) *Ve *locus, formed by two linked genes, *Ve1 *and *Ve2*. To characterize the resistance response mediated by the tomato *Ve *gene, we inoculated two nearly isogenic tomato lines, LA3030 (*ve*/*ve*) and LA3038 (*Ve*/*Ve*), with *V. dahliae*.

**Results:**

We found induction of H_2_O_2 _production in roots of inoculated plants, followed by an increase in peroxidase activity only in roots of inoculated resistant plants. Phenylalanine-ammonia lyase (PAL) activity was also increased in resistant roots 2 hours after inoculation, while induction of PAL activity in susceptible roots was not seen until 48 hours after inoculation. Phenylpropanoid metabolism was also affected, with increases in ferulic acid, *p*-coumaric acid, vanillin and *p*-hydroxybenzaldehyde contents in resistant roots after inoculation. Six tomato *PAL *cDNA sequences (*PAL1 *- *PAL6*) were found in the SolGenes tomato EST database. RT-PCR analysis showed that these genes were expressed in all organs of the plant, albeit at different levels. Real-time RT-PCR indicated distinct patterns of expression of the different *PAL *genes in *V. dahliae*-inoculated roots. Phylogenetic analysis of 48 partial *PAL *cDNAs corresponding to 19 plant species grouped angiosperm *PAL *sequences into four clusters, suggesting functional differences among the six tomato genes, with *PAL2 *and *PAL6 *presumably involved in lignification, and the remaining *PAL *genes implicated in other biological processes.

An increase in the synthesis of lignins was found 16 and 28 days after inoculation in both lines; this increase was greater and faster to develop in the resistant line. In both resistant and susceptible inoculated plants, an increase in the ratio of guaiacyl/syringyl units was detected 16 days after inoculation, resulting from the lowered amount of syringyl units in the lignins of inoculated plants.

**Conclusions:**

The interaction between the tomato and *V. dahliae *triggered a number of short- and long-term defensive mechanisms. Differences were found between compatible and incompatible interactions, including onset of H_2_O_2 _production and activities of peroxidase and PAL, and phenylpropanoid metabolism and synthesis of lignins.

## Background

Verticillium wilt, caused by the vascular fungus *Verticillium dahliae *Kleb., limits the production of a wide range of economically important crops [1]. Once the fungus infects a field, it is very persistent because it colonizes such non-host plants as cereals, which then act as reservoirs for the fungus. Furthermore, the fungus develops resistant structures known as microsclerotia that are capable of survival in the soil for decades. Significant losses are caused by this pathogen, and currently there are no efficient management methods for its control. The *Verticillium *spp. are among the most damaging pathogens threatening cultivation of the tomato (*Solanum lycopersicum*), and are responsible for serious economic losses both in greenhouses and in the field. The cultivation of resistant varieties has proven to be an appropriate strategy for combating plant pathogens because of its efficacy, low cost, and limited environmental impact. Recent developments in molecular biology have made it possible to transfer resistance genes between unrelated species and have revealed that the molecular events in the resistance response elicited from recognition of the pathogen are often conserved among plants of the same family [2].

The only *Verticillium *resistance genes in Solanaceae that are now known are those of the tomato *Ve *locus. This locus is formed by two linked genes, *Ve1 *and *Ve2*, each capable of conferring resistance to different *Verticillium *species. The structure of the *Ve *genes suggests that they code for cell-surface glycoproteins with signals for receptor-mediated endocytosis and with leucine zipper motifs (in *Ve1*) or PEST sequences (in *Ve2*) [3]. Potato plants transformed with either of the *Ve *genes acquired resistance against *Verticillium*, demonstrating that the cell machinery required for the incompatible (no disease) interaction with *Verticillium *is present and functional in other Solanaceae species.

Events in the early stages of a plant's response to an infecting pathogen determine the degree of colonization and the damage caused. An incompatible interaction generally seems to require the presence in the plant of a cognate resistance gene against an avirulence factor of the pathogen [4]. However, incompatibility is probably related more to the timing of induction of defense genes and factors than to qualitative differences in the set of genes expressed when compared to compatible systems [5]. During an incompatible interaction, plant cells respond with such resistance strategies as (i) generation of reactive oxygen species (ROS), (ii) induction of a hypersensitive response, a localized cell-death reaction that confines the infection to its initial location, (iii) expression of pathogenesis-related genes and other toxic peptides, (iv) synthesis of phytoalexins, (v) stabilization of cell walls, and (vi) closure of the stomata [6].

The production of oxygen intermediates during the so-called oxidative burst is characteristic of the defensive response in plants [7]. Increased levels of ROS, notably the superoxide anion (O_2_^-^) and hydrogen peroxide (H_2_O_2_), kill the pathogen or limit colonization by triggering a hypersensitive response in the infected plant tissue [8]. H_2_O_2 _is the most stable oxygen intermediate and is involved in the cross-linking of cell wall components [9], regulation of pathogenesis-related gene expression [10], transduction of the hypersensitive response [7], and killing of invading pathogens [11]. H_2_O_2 _also acts as a signaling molecule in the cellular collapse that occurs during the hypersensitive response, and in systemic acquired resistance [12].

The peroxidases (PODs; EC 1.11.1.7; donor H_2_O_2_-oxidoreductase) are heme-containing enzymes that catalyze the oxidation of different substrates using H_2_O_2_. They also produce ROS as a result of their peroxidative and hydroxylic catalytic cycles [13]. Peroxidases are widely distributed in the plant kingdom [14,15] and are active in such physiological processes as ferulate dimerization [16], phenol oxidation [17] and lignification [18,19]; these mechanisms may be activated in the defensive response against pathogens [20].

Some authors have reported the involvement of peroxidases in the formation of phenylpropanoid dimers using equimolar mixtures of hydroxycinnamic acid [21]. Peroxidases contribute to the construction of the cell wall. These actions include intervention in the possible covalent binding of tyrosine residues from extensin and other cell wall glycoproteins with dimers of hydroxycinnamic acid and *p*-hydroxybenzoic acid bound to pectins or some xylans. Peroxidases are also involved in the biosynthesis of lignins, in the deposition of lignin bound to cell wall glycoproteins, and in the process of suberization [22].

Phenylalanine ammonia lyase (PAL; L-phenylalanine ammonia lyase, EC 4.3.1.5) is the first enzyme in the phenylpropanoid metabolism pathway. PAL catalyzes the deamination of phenylalanine to *trans-*cinnamic acid, the common precursor for the synthesis of all phenol derivatives. A number of studies have reported increased PAL expression and activity in response to environmental stimuli, such as cold [23], wounding [24] and UV-B light [25]. Silencing of *PAL *genes in transgenic tobacco lines inhibits the salicylate production normally seen after tobacco mosaic virus infection, and abolishes systemic acquired resistance [26]. *PAL *over-expression in tobacco leads to production of large amounts of chlorogenic acid, and a marked reduction in sensitivity to infection by the fungus *Cercospora nicotianae *[27].

In higher plants, *PAL *is found as a family of homologous genes. The significance of this diversity is unclear, but is consistent with the complexity of metabolic pathways in phenylpropanoid metabolism. Four *PAL *genes have been described in Arabidopsis [28], five in pine and tomato [29,30], and a total of 16 in the genome of a diploid potato hybrid [31].

The onset of phenylpropanoid metabolism is another crucial defensive mechanism [32]. This results in hydroxycinnamic acids with a characteristic C_6_C_3 _phenylpropane skeleton being produced from the primary metabolite phenylalanine. The functions of the phenylpropanoids are very diverse: some are pigments, others phytoalexins, phytoanticipins, UV-protectants or signals mediating the interaction between plants and microorganisms. Furthermore, some phenylpropanoids can polymerize and form defensive structures, such as lignin [33]. There is strong evidence to suggest that esterification of phenols, such as ferulic or *p*-coumaric acids, to cell walls is a common phenomenon in the expression of resistance [34-36]. It is generally thought that phenols play an important role in the modification of the mechanical properties of cell walls [37], limiting polysaccharide degradation by exogenous enzymes [38,39] and increasing cell wall rigidity by linking polysaccharides and lignin [40].

Lignins are amorphous heteropolymers that result from oxidative coupling of *p*-coumaryl, coniferyl and sinapyl alcohols, forming the subunits H (hydroxyphenyl), G (guaiacyl) and S (syringyl), respectively. Lignins are primarily deposited in cell walls of tissues as tracheids, veins, fibers of xylem and phloem, and schlereids. Lignin composition varies depending on the tissue. For example, lignins type G are predominant in *Arabidopsis *xylem, while in schlerenchyma cells lignins type S are more commonly found [41]. Lignification of cell walls is a key event in resistance against pests in herbaceous or woody plants and resistant genotypes possess a greater accumulation of lignins [42]. Ratios among lignin subunits change after a pathogen attack [43]. Several mechanisms have been postulated to explain the role of lignins in resistance, including sealing of cell walls [38] or direct biocidal effects of phenolic lignin precursors [44].

In this study we examine H_2_O_2 _production, peroxidase and PAL activity, expression of *PAL *genes and lignin accumulation in two nearly isogenic tomato lines after exposure to *V. dahliae*, one line carrying the *Ve *resistance gene and the other not. This characterization of *Ve*-mediated resistance at the molecular level may allow identification of factors which, after induction, can trigger resistance responses in tomato and in other Solanaceae.

## Results

### H_2_O_2 _content

**Figure 1:**
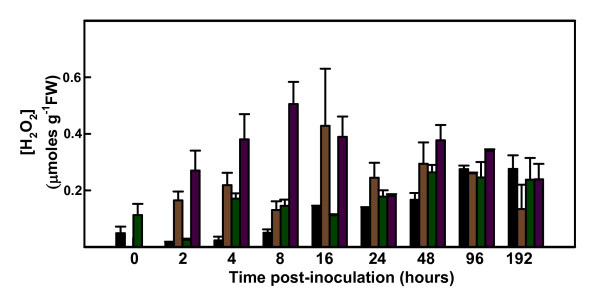
**H_2_O_2 _content of roots of control and inoculated susceptible and resistant tomato plants**. Measurement of H_2_O_2 _content of roots of control and inoculated LA3030 (susceptible) and LA3038 (resistant) tomato plants using the xylenol orange method. Control LA 3030 (black bars); inoculated LA 3030 (brown bars); control LA 3038 (green bars); inoculated LA3038 (purple bars).

H_2_O_2 _production in roots of control and *V. dahliae*-inoculated resistant and susceptible tomato plants was measured using the xylenol orange method, which is very sensitive for the detection of low levels of soluble hydroperoxides. Definite variations in the production of H_2_O_2 _were found in both tomato lines, whether inoculated or not (Figure 1). At 2 hours post-inoculation (hpi) a dramatic increase in H_2_O_2 _content was observed in the roots of inoculated resistant plants, with a maximum peroxide concentration found at 8 hpi, when H_2_O_2 _content was three times higher than that in resistant controls or in inoculated susceptible plants. In resistant plants, a second lesser increase was detected between 24 hpi and 48 hpi. In inoculated susceptible plants, a single increase was observed starting at 2 hpi, reaching a peak at 16 hpi. At the final time point monitored, 192 hpi, H_2_O_2 _content was similar in control and inoculated roots of the two lines.

### Peroxidase (POD) activity

**Figure 2:**
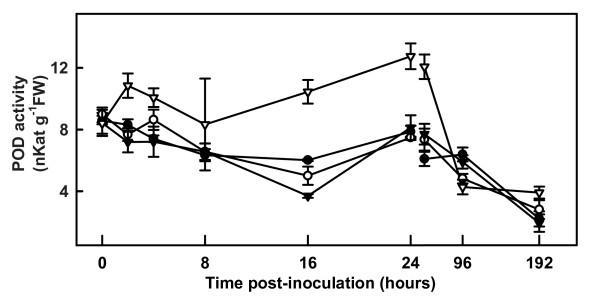
**Peroxidase activity in roots of control and inoculated susceptible and resistant tomato plants**. Measurement of peroxidase (POD) activity in the roots of control and inoculated LA3030 (susceptible) and LA3038 (resistant) tomato plants using 4-methoxynaphthol as a substrate. Control LA3030 (closed circles); inoculated LA3030 (open circles); control LA3038 (closed triangles); inoculated LA3038 (open triangles). Note the gap and change of scale on the X-axis after 24 h.

Because the binding of phenolic compounds to the cell wall is mediated by peroxidases and, during plant-pathogen interactions, this binding occurs at the expense of a massive generation of H_2_O_2 _[27], we monitored peroxidase activity. In our experiments, the inoculation of resistant plants with *V. dahliae *led to a rapid increase in peroxidase activity in roots, detectable at 2 hpi (Figure 2). Similar activity levels were then maintained from 4 and 8 hpi; subsequently another increase in peroxidase activity was observed, reaching a maximum between 24 hpi and 48 hpi. No appreciable changes were detected in inoculated susceptible roots throughout the experiment. At the end of the study period (192 hpi), similar peroxidase activities were found in susceptible, resistant, inoculated and control samples.

### Phenylalanine ammonia lyase (PAL) activity

**Figure 3:**
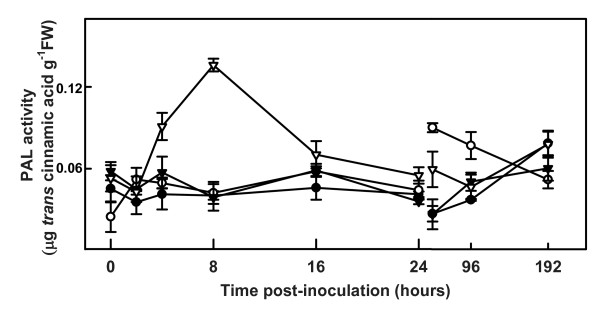
**Phenylalanine ammonia lyase activity in roots of control and inoculated susceptible and resistant tomato plants**. Measurement of phenylalanine ammonia lyase (PAL) activity in roots of control and inoculated LA3030 (susceptible) and LA3038 (resistant) tomato plants using L-phenylalanine as a substrate. Control LA3030 (closed circles); inoculated LA3030 (open circles); control LA3038 (closed triangles); inoculated LA3038 (open triangles). Note the gap and change of scale on the X-axis after 24 h.

The crucial role of PAL within plant secondary metabolism reflects its function as a catalyst for the first step of phenylpropanoid metabolism. We found an increase in PAL activity in roots of inoculated resistant plants, detectable at 4 hpi, with a maximum at 8 hpi, when activity was approximately 3-fold higher than initial values (Figure 3). After 16 hpi, PAL activity decreased, and by 24 hpi it was identical to that in controls and in inoculated susceptible plants. In inoculated susceptible plants, PAL activity peaked at 48 hpi, at around 70% of the maximum value seen in inoculated resistant plants, although it was 50% higher than in inoculated resistant plants at the same time point. At 192 hpi, PAL activity was similar in roots of both lines, whether inoculated or not.

### RT-PCR of *PAL *genes from different organs

**Figure 4:**
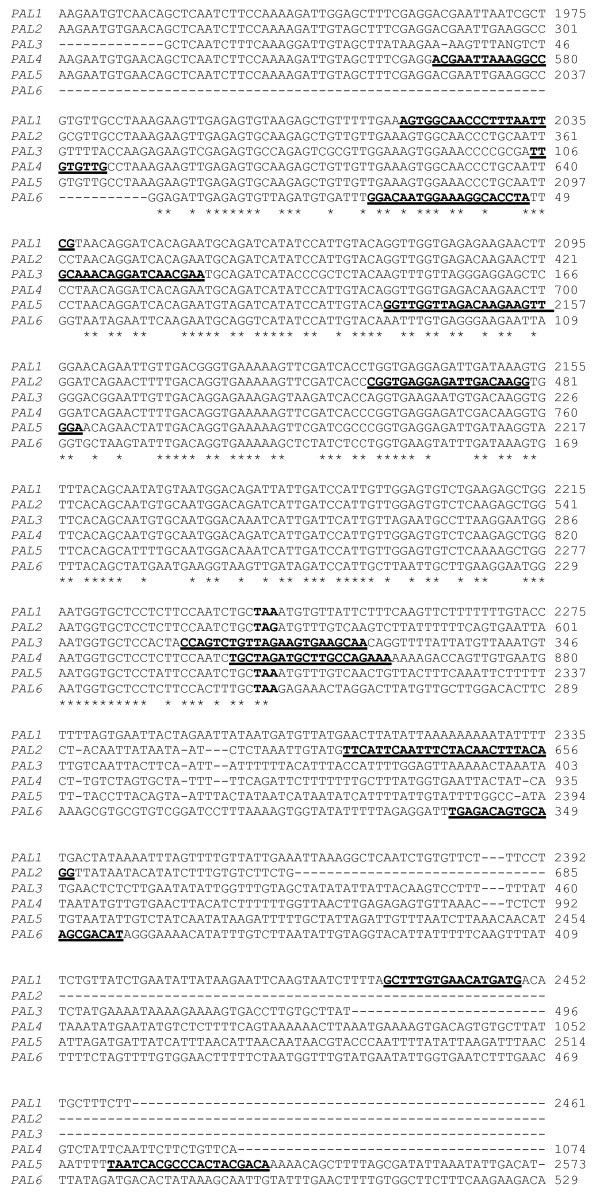
**Alignment of the tomato *PAL *gene cDNA sequences**. Alignment of the tomato *PAL *gene cDNA sequences. The stop codons are shown in boldface. Regions underlined and in boldface were used for the design of primers for RT-PCR experiments.

After detection of the rapid increase in PAL activity in roots of inoculated resistant plants, we studied possible differences in the level of expression of different tomato *PAL *genes. An extensive search was performed in the NCBI and TIGR databases for cDNA and contig sequences of the tomato *PAL *genes. As a result, the sequences of 6 tomato *PAL *genes that differed in their noncoding 3' ends were determined (Figure 4). The *β*-tubulin gene was chosen as a constitutive gene for quantification experiments.

**Figure 5:**
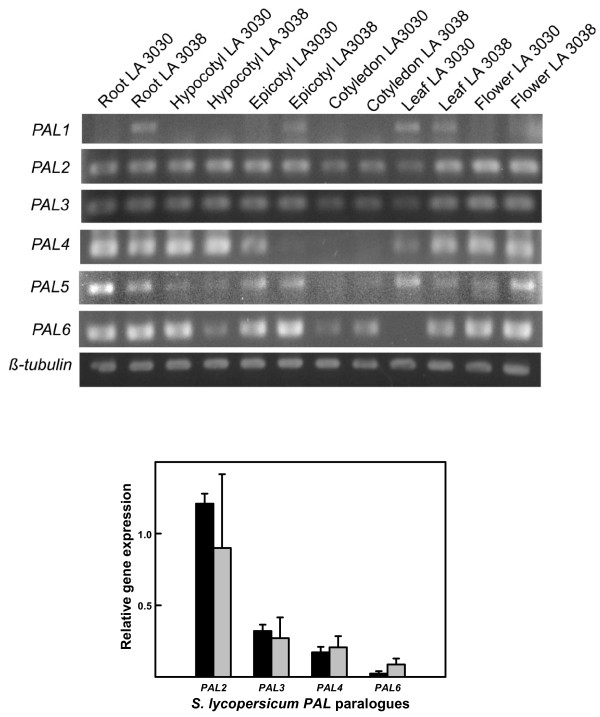
**Analysis of expression of *PAL *genes**. Figure 5A. In-gel RT-PCR expression of the *PAL *genes in various organs of susceptible (LA3030) and resistant (LA3038) tomato lines, compared with expression in the same organs of the constitutive control gene *β-tubulin*. Figure 5B. Real-time RT-PCR analysis of the levels of expression of the genes *PAL2*, *PAL3*, *PAL4 *and *PAL6 *in roots of susceptible LA 3030 (black bars) and resistant LA3038 (gray bars) tomato plants.

Following RNA extraction from roots, hypocotyls, epicotyls, cotyledons, leaves and flowers from resistant and susceptible tomato plants, the corresponding cDNAs were synthesized and PCR amplifications carried out (Figure 5A). Amplicons from *PAL2*, *PAL3, PAL4 *and *PAL6 *were clearly visualized on ethidium bromide-stained agarose gels when 5 ng RNA was used as the starting material. In contrast, for visualization of amplicons, 200 ng RNA was required for *PAL5 *and 600 ng RNA for *PAL1 *in all organs analyzed. This finding indicated the existence of different levels of expression of the *PAL *genes in the tomato. In summary, expression of the six *PAL *genes could be detected in all organs studied, albeit at different levels, with lower expression levels for *PAL5 *and lowest for *PAL1*.

### Real-time RT-PCR analysis of *PAL2*, *PAL3*, *PAL4 *and *PAL6 *in tomato roots

We next analyzed root samples from LA3030 and LA3038 tomato plants using real-time RT-PCR. Because of the extremely low levels of expression of the *PAL1 *and *PAL5 *genes, which led to inconsistent results from RT-PCR, we considered only the four remaining *PAL *genes. The results showed slight differences in expression among these genes (Figure 5B). The highest relative expression value was found for *PAL2*, followed by *PAL3 *and *PAL4*, which had similar expression levels. The lowest level of expression was found for *PAL6*. This was the only gene that showed any significant difference between LA3030 and LA3038 plants: its expression level was three times higher in LA3038, the resistant plant.

### Real-time RT-PCR analysis of the main *PAL *genes in *V. dahliae*-inoculated roots

**Figure 6:**
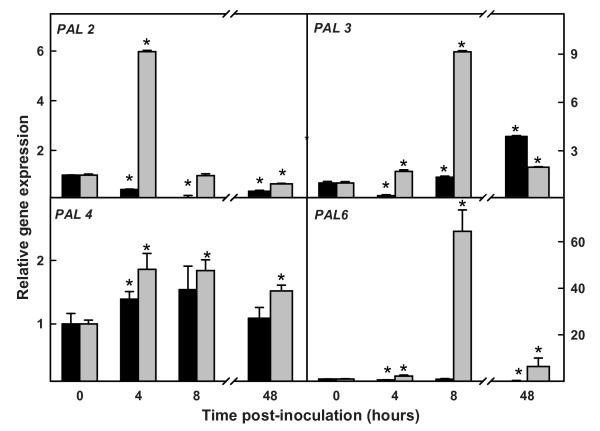
**RT-PCR of *PAL2*, *PAL3*, *PAL4 *and *PAL6 *in roots of susceptible and resistant tomato plants**. RT-PCR analysis of relative levels of expression of the genes *PAL2*, *PAL3*, *PAL4 *and *PAL6 *in roots of susceptible LA3030 (black bars) and resistant LA3038 (gray bars) tomato plants following inoculation with *V. dahliae*. Bars labeled with an asterisk (*) are significantly different (*p *< 0.05).

We then used real-time RT-PCR to quantify the expression of *PAL2*, *PAL3*, *PAL4 *and *PAL6 *at specific intervals following inoculation of resistant and susceptible tomato roots with *V. dahliae *(Figure 6).

*PAL2 *showed maximum expression in inoculated resistant plants at 4 hpi and then decreased to a level similar to the controls by 8 hpi. The maximum level was 6 times higher than that in the control plants of both lines. In contrast, in inoculated susceptible plants, *PAL2 *showed a significant decrease in expression as early as 4 hpi (*P*-value < 0.05).

*PAL3 *expression in inoculated resistant plants showed a detectable increase at 4 hpi, with the maximum level reached at 8 hpi. The maximum level was 9 times higher than that in the controls and in inoculated susceptible plants. In inoculated susceptible plants, there was an increase in gene expression of PAL3 at 48 hpi, a level 3.5 times higher than in the controls and double that seen in the inoculated resistant plants at that time. Interestingly, this was the only increase in expression of a *PAL *gene seen in susceptible plants.

*PAL4 *expression was slightly higher in all the inoculated resistant plants than in the inoculated susceptible plants.

*PAL6 *showed a dramatic increase in expression in the inoculated resistant plants at 8 hpi, with a level of expression approximately 60 times higher than that seen in the control plants of both lines and in the inoculated susceptible plants.

### Phylogenetic relationships among plant *PAL *genes

**Figure 7:**
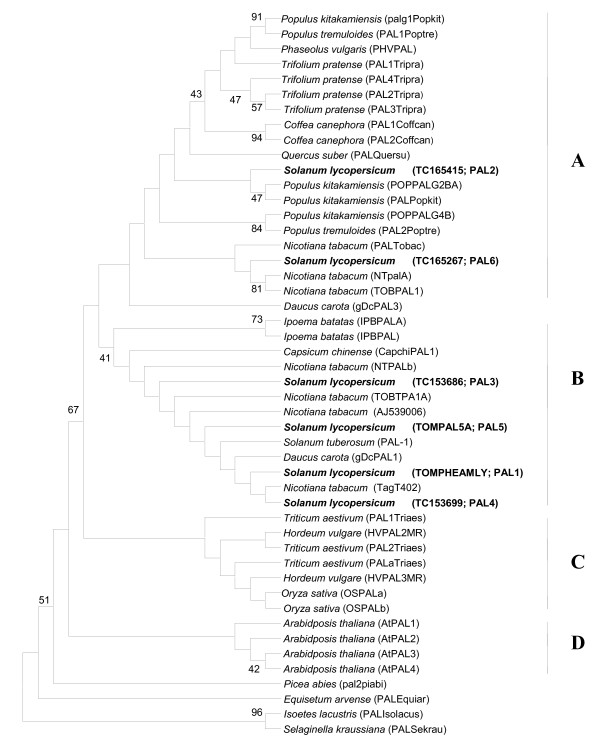
**Phylogenetic analysis of *PAL *nucleotide sequences**. Strict consensus tree of the 814 most parsimonious trees of 19 plant species based on 48 *PAL *nucleotide sequences restricted to 116 nucleotides from the 3' end of the coding region (CI = 0.475, RI = 0.756, RCI = 0.359). Numbers in gene names indicate multiple homologues from the same plant species.

The sequences of 48 *PAL *genes, belonging to 19 plant species, stored in the GenBank database (Table 1) were retrieved and compared to the six tomato cDNA sequences. A 116-nucleotide sequence from the 3' end of the coding region was chosen for the comparison. The resulting maximum parsimony phylogenetic tree (Figure 7) was rooted in the sequences from the most ancient species, the pteridophyte *Isoetes lacustis *and the spikemoss *Selaginella kraussiana*. Sequences from all angiosperm species were grouped in four different clusters (A, B, C and D), with the *Equisetum arvense *(Pteridophyta) and *Picea abies *(Gymnosperma) sequences in two independent branches. The 6 *PAL *genes from *S. lycopersicum *were placed in two different clusters, A and B. In cluster A, besides the tomato genes *PAL2 *and *PAL6*, most of the gene sequences belonged to woody plants (*Populus kitakamiensis*, *Populus tremuloides, Coffea canephora *and *Quercus suber*); though sequences from *Trifolium pratense*, *Nicotiana tabacum *and *Daucus carota *were also included in this cluster. The four remaining tomato genes (*PAL1, PAL3, PAL4 *and *PAL5*) were placed in cluster B, together with sequences from other dicotyledonous species including *N. tabacum*, *D. carota*, *Solanum tuberosum*, *Capsicum chinense *and *Ipoema batatas*. A third cluster, C, was composed of the *PAL *sequences from monocotyledonous species (*Hordeum vulgare*, *Oryza sativa *and *Triticum aestivum*) and a fourth cluster, D, was exclusively formed by the four *PAL *sequences from *A. thaliana*.

**Table 1:** Listing of phenylalanine ammonia lyase *(PAL) *genes included in the phylogenetic analysis.

Plant species	Identification name	Accession number	Plant species	Identification name	Accession number
*A. thaliana*	*AtPAL1*	AY303128	*N. tabacum*	*PALTobac*	X78269
*A. thaliana*	*AtPAL2*	AY303129	*N. tabacum*	*TagT402*	(1)
*A. thaliana*	*AtPAL3*	AY528562	*N. tabacum*	*TOBPAL1*	D17467
*A. thaliana*	*AtPAL4*	AY303130	*N. tabacum*	*TOBTPA1A*	M84466
*C. chinense*	*CapchiPAL1*	AF081215	*O. sativa*	*OSPALa*	XM_473192
*C. canephora*	*PAL1Cofcan*	AF460203	*O. sativa*	*OSPALb*	XM_466843
*D. carota*	*gDcPAL1*	D85850	*P. abies*	*pal2piabi*	AM293549
*D. carota*	*gDcPAL3*	AB089813	*P. kitakamiensis*	*palg1Popkit*	D30656
*E. arvense*	*PALEquiar*	AY803283	*P. kitakamiensis*	*PALPopkit*	D30657
*H. vulgare*	*HVPAL2MR*	Z49145	*P. kitakamiensis*	*POPPALG2BA*	D43802
*H. vulgare*	*HVPAL3MR*	Z49146	*P. kitakamiensis*	*POPPALG4B*	D43803
*I. batatas*	*IPBPALA*	D78640	*P. tremuloides*	*PAL1Poptre*	AF480619
*I. batatas*	*IPBPAL*	M29232	*P. tremuloides*	*PAL2Poptre*	AF480620
*C. canephora*	*PAL2Coffcan*	AF460204	*Ph. vulgaris*	*PHVPAL*	M11939
*I. lacustris*	*PALIsolacus*	AY803281	*Q. suber*	*PALQuersu*	AY443341
*S. lycopersicum*	*PAL1*	TC153702	*S. kraussiana*	*PALSekrau*	AY803282
*S. lycopersicum*	*PAL2*	TC165415	*S. tuberosum*	*PAL-1*	X63103
*S. lycopersicum*	*PAL3*	TC153686	*T. aestivum*	*PAL1Triaes*	X99705
*S. lycopersicum*	*PAL4*	TC153699	*T. aestivum*	*PAL2Triaes*	X99725
*S. lycopersicum*	*PAL5*	TC153688	*T. aestivum*	*PALaTrieas*	AY005474
*S. lycopersicum*	*PAL6*	TC165267	*T. pratense*	*PAL1Tripra*	DQ073809
*N. tabacum*		AJ539006	*T. pratense*	*PAL2Tripra*	DQ073810
*N. tabacum*	*NTpalA*	AB008199	*T. pratense*	*PAL3Tripra*	DQ073808
*N. tabacum*	*NTPALb*	AB008200	*T. pratense*	*PAL4Tripra*	DQ073811

(1) Reference [58]

### Phenolic compounds in roots

To investigate the effects of *V. dahliae *inoculation on the contents of phenolic compounds, root samples were collected and analyzed. Some phenols, notably hydroxycinnamic acids, are involved in cell wall reinforcement, which enhances plant resistance to fungal colonization of the vascular system. Therefore, we determined whether there were any changes in the levels of specific phenols related to cell wall reinforcement. Two hydroxycinnamic acids, ferulic acid and *p*-coumaric acid, and their respective benzaldehydes, vanillin and *p*-hydroxybenzaldehyde, were analyzed using reverse-phase HPLC. Ferulic acid levels showed only a small increase in inoculated resistant plants at 2 hpi, while *p*-coumaric acid level showed an increase between 16 and 96 hpi. The vanillin level was significantly higher in inoculated resistant plants between 96 and 192 hpi. *p*-Hydroxybenzaldehyde was found in inoculated resistant roots between 24 and 192 hpi. No changes in levels of these phenols were observed in susceptible plants (Table 2).

**Table 2:** HPLC analyses of phenylpropanoids.

		Phenolic content (μg g^-1 ^FW)							
		
		Ferulic acid		*p-*Coumaric acid		Vanillin		*p*-Hydroxybenzaldehyde	
		
		LA3030	LA3038	LA3030	LA3038	LA3030	LA3038	LA3030	LA3038
2 hpi	C	2.63 ± 0.36	2.59 ± 0.01	0.22 ± 0.02	0.54 ± 0.01	0.14 ± 0.01	0.09 ± 0.01	0.49 ± 0.03	0.48 ± 0.01
	I	3.50 ± 0.01	5.16 ± 0.29*	0.41 ± 0.06	0.27 ± 0.01	0.24 ± 0.01	0.26 ± 0.01	0.40 ± 0.01	0.42 ± 0.05
4 hpi	C	3.39 ± 1.12	2.68 ± 0.01	0.43 ± 0.05	0.33 ± 0.09	0.20 ± 0.01	0.21 ± 0.01	0.43 ± 0.04	0.38 ± 0.02
	I	2.31 ± 1.63	3.34 ± 0.93	0.38 ± 0.15	0.37 ± 0.07	0.20 ± 0.01	0.24 ± 0.01	0.37 ± 0.01	0.43 ± 0.01
8 hpi	C	2.75 ± 0.49	2.91 ± 0.14	0.42 ± 0.02	0.48 ± 0.01	0.21 ± 0.01	0.20 ± 0.01	0.39 ± 0.04	0.37 ± 0.01
	I	2.34 ± 1.62	3.30 ± 0.93	0.51 ± 0.09	0.44 ± 0.18	0.20 ± 0.09	0.24 ± 0.05	0.38 ± 0.08	0.48 ± 0.02
16 hpi	C	1.89 ± 0.12	3.42 ± 0.12	0.20 ± 0.19	0.46 ± 0.01	0.16 ± 0.01	0.15 ± 0.01	0.25 ± 0.09	0.38 ± 0.01
	I	3.22 ± 0.22	2.92 ± 0.13	0.35 ± 0.14	1.00 ± 0.29*	0.20 ± 0.01	0.19 ± 0.02	0.34 ± 0.05	0.54 ± 0.03
24 hpi	C	2.94 ± 0.03	2.95 ± 0.43	0.10 ± 0.02	0.51 ± 0.05	0.14 ± 0.01	0.23 ± 0.01	0.32 ± 0.07	0.33 ± 0.06
	I	3.68 ± 0.17	3.56 ± 0.31	0.41 ± 0.11	1.01 ± 0.12*	0.22 ± 0.03	0.27 ± 0.01	0.37 ± 0.07	0.85 ± 0.07*
48 hpi	C	2.85 ± 0.03	3.61 ± 1.39	0.63 ± 0.46	0.60 ± 0.23	0.15 ± 0.01	0.20 ± 0.01	0.35 ± 0.07	0.49 ± 0.02
	I	1.88 ± 0.95	3.33 ± 1.29	1.01 ± 0.68*	0.92 ± 0.40*	0.17 ± 0.01	0.27 ± 0.06	0.52 ± 0.04*	0.97 ± 0.01*
96 hpi	C	3.65 ± 0.25	3.25 ± 0.21	0.40 ± 0.02	0.46 ± 0.30	0.23 ± 0.01	0.21 ± 0.02	0.31 ± 0.03	0.44 ± 0.02
	I	4.85 ± 0.09	4.57 ± 0.50	1.51 ± 0.34*	0.61 ± 0.14*	0.23 ± 0.01	0.43 ± 0.02*	0.57 ± 0.04*	1.61 ± 0.01*
192 hpi	C	3.31 ± 0.01	3.44 ± 0.17	0.57 ± 0.27	0.40 ± 0.35	0.20 ± 0.01	0.21 ± 0.03	0.38 ± 0.01	0.37 ± 0.08
	I	3.46 ± 0.26	4.48 ± 1.68	0.66 ± 0.46	0.45 ± 0.22	0.22 ± 0.05	0.34 ± 0.02*	0.38 ± 0.01	0.80 ± 0.07*

HPLC analysis of ferulic acid, *p*-coumaric acid, vanillin and *p*-hydroxybenzaldehyde contents in the cell wall-bound fraction of control and *V. dahliae*-inoculated resistant (LA3038) and susceptible (LA3030) tomato roots. The results are the mean values of three independent assays. Hpi: hours post-inoculation; C: control plants; I: inoculated plants; FW: fresh weight. Means followed by * are significantly different from the control group mean for that time point (*P *< 0.05).

### Lignin content, monomer composition and cross-linking in roots

The total lignin content of cell walls, as measured by acetyl bromide is shown in Table 3. An increase in lignin content was observed in inoculated resistant roots at 16 and 28 days post-inoculation (dpi). In inoculated susceptible roots, the increase was not found until 28 dpi. Nitrobenzene oxidation in an alkaline medium degrades lignins forming *p*-hydroxybenzaldehyde from hydroxyphenyl (H), vanillin from guaiacyl (G) and syringyl aldehyde from syringyl (S). Table 3 also shows the relative monomeric composition of cell walls of susceptible and resistant roots at 16 and 28 dpi, calculated after nitrobenzene oxidation. In inoculated plants at 16 dpi, there was an increase in percentage of G-units at the expense of S groups. The increase in G groups produces higher G/S ratios at 16 dpi after *V. dahliae *inoculation. At 28 dpi, the G/S ratio was the same among control and inoculated resistant or susceptible plants, but an increase in H subunits was detected.

**Table 3:** Changes in lignin content following *V. dahliae *inoculation.

			Lignin content (μg/mg CW)	Relative monomeric composition (%)			
				
				H-units	G-units	S-units	G/S ratio
	LA3030	Control	11.2 ± 0.6^a^	25	52	33	2.2
16 dpi		Inoculated	13.0 ± 0.9^a^	26	62	12	5.5
	LA3038	Control	10.2 ± 0.8^a^	29	53	17	2.9
		Inoculated	18.6 ± 0.2^b^	30	59	10	5.9
	LA3030	Control	10.6 ± 0.2^a^	28	58	14	4.1
28 dpi		Inoculated	19.5 ± 0.3^b^	32	55	12	4.7
	LA3038	Control	13.5 ± 0.5^a^	28	58	13	4.5
		Inoculated	17.3 ± 0.7^b^	31	56	13	4.4

Lignin content, measured by acetyl bromide, and monomeric composition, revealed by analysis of nitrobenzene oxidation, of root cell walls products from control and *V. dahliae*-inoculated LA3030 (susceptible) and LA3038 (resistant) tomato plants at 16 and 28 days post-inoculation. H-units: hydroxyphenyl units; G-units: guaiacyl units; S-units: syringyl units; CW: cell walls; dpi: days post-inoculation. Values followed by the same superscript letter are not significantly different from controls (P < 0.05)

The possibility of other qualitative changes in composition and structure of cell walls was assessed using the thioacidolysis degradative method. Fragments resulting from thioacidolysis were identified by GC/MS. We found primarily thioethylated monomers (erythro- and threo- isomers) resulting from aryl-glycerol-β-aryl ether structures derived from coniferyl and sinapyl alcohols and from their respective cinnamyl aldehydes. Terminal *O-4 *structures from coniferyl alcohol, *p*-coumaric acid, ferulic acid, vanillin and dihydroconiferyl alcohol were also found. Table 4 shows the values of ionic current for all fragments. A general increase in abundance of a large majority of fragments was found at 28 dpi in inoculated plants, resistant or not, in concordance with the results from acetyl bromide. At 28 dpi, inoculated resistant plants had significant increases in *β-O-4 *monomers and also showed a 3.3-fold increase in coniferyl aldehyde, a 8.4-fold increase in the terminal O-4 monomers of dihydroconiferyl alcohol, and a 4.2 increase in vanillin.

**Table 4:** Cell wall thiacidolysis analyses after *V. dahliae *inoculation.

			*β-O-4*				*O-4*end				
				
			CA	CAd	SA	SAd	DHCA	pCA	CA	V	FA
	LA3030	C	1438.9	18.1	998.4	49	Tr	2.1	8.5	3.2	10.1
16 dpi		I	1363.8	40.1	719.5	32.2	Tr	2.0	7.9	4.3	148.1
	LA3038	C	1307.9	18.3	916.7	21.0	Tr	2.1	9.8	3.1	4.1
		I	1240.2	40.3	659.2	18.0	8.0	2.0	18.3	6.4	287.3
	LA3030	C	1499.2	52.3	702.0	83	0.9	1.9	18.0	4.1	120.8
28 dpi		I	1707.2	59.8	1351.9	65.0	1.2	3.4	15.9	5.1	158.9
	LA3038	C	1213.9	40.2	973.7	67.0	0.7	2.2	40.1	3.9	162.1
		I	2173.4	133.5	1703.2	97.0	5.9	4.1	63.7	16.2	385.1

Monomeric degradation products obtained by thioacidolysis of root cell walls from control and *V. dahliae*-inoculated LA3030 (susceptible) and LA3038 (resistant) tomato plants at 16 and 28 days post-inoculation. Values are given in Total Ionic Current (TIC) × 10^-8^. SD values were within 5%. β-*O*-4 represents the amount of monomers linked by β-*O*-4 bonds. *O*-4-end represents the amount of *O*-4-linked end monomers. C: control plants; I: inoculated plants; CA: coniferyl alcohol; CAd: coniferylaldehyde; SA: sinapyl alcohol; SAd: sinapylaldehyde; DHCA: dihydroconiferyl alcohol; pCA: p-coumaric acid; V: vanillin, FA: ferulic acid; dpi: days post-inoculation; Tr: trace.

Comparing the ratios among different groups of monomers, considering their chemical nature and type of bond, at 16 dpi an increase in the ratio guaiacyl/syringyl was found in inoculated plants from around 1.4 to more than 2.0 (Table 5). At 16 dpi, G/S ratios were lower than those found after nitrobenzene oxidation, in agreement with the higher level of G groups of the condensed nucleus of lignins, as compared to the linear fraction susceptible to be degraded by thioacidolysis. This increase disappeared at 28 dpi, when ratios in both control and inoculated plants were similar. The ratio between *β-O-4 *and *O-4 *terminal monomers, indicating the level of polymerization of the linear fraction of lignins, decreased at 16 dpi from values close to 100 in the controls to 13.1 in inoculated susceptible and 6.1 in inoculated resistant plants. This decrease in the ratio was primarily due to a dramatic increase in *O-4 *terminal groups, particularly of ferulic acid, in the inoculated plants.

**Table 5:** Relationships among monomeric degradation products after thioacidolysis.

			ΣG	ΣS	G/S ratio	Σ*β-O-4*	Σ *O-4*	*β-O-4/O-4*
	LA3030	Control	1497	1047	1.4	2504	24	104.8
16 dpi		Inoculated	1572	752	2.1	2156	162	13.3
	LA3038	Control	1356	938	1.5	2264	19	118.5
		Inoculated	1617	677	2.4	1958	322	6.1
	LA3030	Control	1712	1435	1.2	2986	146	20.5
28 dpi		Inoculated	1948	1417	1.4	3184	195	16.4
	LA3038	Control	1477	1041	1.4	2295	209	11.0
		Inoculated	2809	1800	1.6	4107	475	8.7

Relationships among the monomeric degradation products resulting from thioacidolysis of root cell walls from control and *V. dahliae*-inoculated LA3030 (susceptible) and LA3038 (resistant) tomato plants at 16 and 28 days post-inoculation. β-*O*-4 represents the amount of monomers linked by β-*O*-4 bonds. *O*-4-end represents the amount of *O*-4-linked end monomers. G: guaiacyl; S: syringyl dpi: days post-inoculation.

Increases of the aldehydes, vanillin and cinnamaldehydes, detected by thioacidolysis were confirmed by FT-IR spectroscopy analyses. The FT-IR spectroscopy of root cell walls showed an absorption band at 1650 cm^-1 ^(Table 6). This band is clearly attributable to the C = O stretching vibration of conjugated/aromatic aldehydes, in which the carbonyl oxygen atom sustains either intramolecular or intermolecular H-bonds [43]. At 16 dpi, inoculated plants showed an increased level of conjugated/aromatic aldehydes in cell walls, which increased further at 28 dpi.

**Table 6:** Fourier transform infrared spectroscopy analysis

		Relative peak area (%)							
		
		LA3030				LA3038			
			
		16 dpi		28 dpi		16 dpi		28 dpi	
**Functional group**	**Peak wavenumber**	**C**	**I**	**C**	**I**	**C**	**I**	**C**	**I**

-OH-	3436 cm^-1^	100	100	100	100	100	100	100	100
-CH_2_-	2921 cm^-1^	53.5	53.4	54.7	54.7	55.0	57.0	55.6	60.6
-CO- (non conjugated)	1734 cm^-1^	62.3	61.8	64.2	54.2	61.2	59.2	63.6	55.7
-CO- (conjugated)	1650 cm^-1^	70.1	85.4	69.8	107.8	75.9	95.2	80.9	102.3

Fourier transform infrared spectroscopy analysis of root cell walls from control and *V. dahliae*-inoculated LA3030 (susceptible) and LA3038 (resistant) tomato plants at 16 and 28 days post-inoculation, with assignment of peak wave number to functional groups. C: control plants; I: inoculated plants; dpi: days post-inoculation

## Discussion

One of the objectives of this study was to monitor any variations in H_2_O_2 _content and peroxidase and PAL enzyme activities during the tomato-*V. dahliae *interaction. Both enzymes are frequently considered to be key players in the development of plant resistance against pathogens [45]. Their coordinated actions results in changes in the relative amounts of phenylpropanoid compounds, commonly regarded as defensive compounds themselves [46]. To analyze this, two nearly isogenic tomato lines differing in the presence of the *Ve *gene conferring resistance to *Verticillium *were used.

H_2_O_2 _production is one of the markers of the oxidative burst that is one of the most rapid events associated with the hypersensitive response in plant-pathogen interactions [12,47]. After *V. dahliae *inoculation of resistant tomato plants, we observed a rapid increase in H_2_O_2 _content in roots at 2 hpi, with a second smaller peak at 48 hpi. The increase in H_2_O_2 _content was slightly delayed in roots of susceptible plants, reaching its peak at 16 hpi. The rapid increase seen in resistant plants is probably related to several known defense mechanisms. H_2_O_2 _has been proposed to act directly as a toxic compound for microbes [11]; to contribute to cell wall reinforcement in plants [48]; and to be responsible for lipid peroxidation and salicylic acid synthesis [49]. In addition, H_2_O_2 _may also play a role in the signal transduction cascade, triggering the coordinate expression of different genes involved in the defensive response, such as those responsible for the hypersensitive response or for the synthesis of pathogenesis-related proteins [50,51]. The second increase in H_2_O_2 _content seen in resistant roots at 48 hpi may reflect the onset of a systemic response, and is in agreement with the timing of systemic pathogenesis-related *(PR) *gene expression and the beginning of salicylic acid accumulation in the tomato [52]. When we monitored changes in peroxidase activity following *V. dahliae *inoculation, we found a small early increase in roots of resistant plants. By 96 hpi, peroxidase activity was the same as in the control samples. Notably, the similar induction of H_2_O_2 _production in susceptible and resistant plants was not paralleled by comparable increases in peroxidase activity in susceptible plants. Peroxidase activity is the result of the action of a large number of enzymes with similar functions. H_2_O_2 _detoxification in roots of resistant plants is probably due to simultaneous actions of peroxidases and catalase, while the latter is probably predominant in susceptible plants. In any case, H_2_O_2 _detoxification is an efficient mechanism in both resistant and susceptible roots. There was a general decline in peroxidase activity over time in all samples, probably related to the aging processes.

Considering the essential role of phenylalanine ammonia lyase (PAL) in phenolic metabolism, we decided to determine any changes in PAL activity during the infection process. PAL catalyzes the first step in the metabolic route responsible for the synthesis of a vast array of plant compounds based on a phenylpropanoid skeleton Peak PAL activity was observed in roots of inoculated resistant plants at 8 hpi, with a later second minor increase. In susceptible plants, there was an increase in PAL activity between 48 and 96 hpi. Considered together, these results indicate a possible correlation between H_2_O_2 _content and PAL activity in roots of resistant plants. Unlike resistant plants, the increase in H_2_O_2 _content in susceptible plants did not result in an increase in PAL activity until 48 hpi. Also, a delay appeared to exist between the production of H_2_O_2 _and the activation of the *PAL *genes in susceptible plants compared to resistant plants. Contradictory findings regarding the activation of the *PAL *genes by H_2_O_2 _have been published showing that generation of H_2_O_2 _did not induce the expression of the *PAL *genes in bean cell cultures [7], but 5 mM H_2_O_2 _induced the expression of *PAL *genes in *Arabidopsis *cell cultures [54]. Our results seem to support the involvement of H_2_O_2 _in the induction of the expression of the *PAL *genes in resistant tomato plant roots, although this effect was less apparent in susceptible plants.

Using RT-PCR, we attempted to detect any changes in gene expression to identify which *PAL *genes are responsible for the observed increases in enzymatic activity. *PAL *is a multigenic system composed of a variable number of highly homologous genes. We found sequences corresponding to at least 6 different *PAL *genes that differed in their noncoding 3' ends in the SolGenes tomato EST database.

In our first approach, we were able to detect expression of all six *PAL *genes in tomato roots, cotyledons, hypocotyls, epicotyls, leaves and flowers using RT-PCR, although some differences in the levels of expression were observed.

To assess whether the differences in PAL activity in *V. dahliae*-challenged roots from resistant and susceptible tomato lines were due to a coordinated increase in the expression of the different *PAL *genes, or whether there was differential regulation of these genes, we assessed changes in expression of *PAL *genes in inoculated roots using real-time RT-PCR. Because *PAL1 *and *PAL5 *amplification from root cDNA samples was barely detectable after even 30 PCR cycles, we considered only the remaining *PAL *genes. This is somewhat contradictory with a previous report in which the expression of individual tomato *PAL *genes was analyzed [30]; in that paper, most of the findings referred to *PAL1 *and *PAL5*. In more recent works, a predominant expression of *PAL5 *has been detected in tomato roots and leaves using RT-PCR [55,56]. In our RT-PCR study, *PAL2 *was the most abundant transcript, followed by *PAL3*, *PAL4 *and *PAL6*.

Our results revealed differing patterns of expression of the *PAL *genes following *V. dahliae *inoculation. Most of the total increase in *PAL *expression in resistant roots in the first 4 hours after inoculation was from increased *PAL2 *transcription in the initial moments of the interaction. By 8 hpi, however, expression of *PAL2 *had returned to its original level and *PAL3 *and *PAL6 *expression was increased in the roots of resistant plants. *PAL3 *had increased expression at 48 hpi in roots of susceptible plants, coincident with the increase in PAL activity in roots of inoculated susceptible plants. Without ruling out possible posttranscriptional changes, most of the increase in PAL activity in roots of infected susceptible plants seems to have come from the increase in *PAL3 *expression.

*PAL6 *showed a dramatic increase in expression at 8 hpi, which together with the increase in *PAL3 *expression, could explain the maximum peak in PAL activity observed at this time point in inoculated resistant roots. This may indicate that *PAL6 *was the main gene responsible for the increase in PAL activity detected in roots of resistant plants at 8 hours after *V. dahliae *inoculation.

Apart from the 4-fold change in *PAL3 *expression at 48 hpi, no clear changes in *PAL *expression were detected in the compatible interaction. Interestingly, the only clear change in *PAL *expression observed at 48 hpi in the incompatible interaction corresponded to a different gene, *PAL6*, with a 6-fold increase compared to the control. This change in expression might explain the second minor increase in PAL activity found in roots of resistant plants at 48 hpi and could indicate the establishment of a systemic response.

Any differences in function or substrate affinity among the different PAL proteins in the tomato are not defined at this time. Kinetic parameters of the *Arabidopsis thaliana*

PAL isoforms AtPAL1, 2 and 4 indicate that all three followed standard Michaelis-Menten kinetics. However, AtPAL3 was estimated to have a catalytic efficacy 500- to 1000-fold lower than that of the other PAL isoforms, based on its higher *K*_M _and very low *K*_cat _values [28].

We next decided to analyze the structural relationship of tomato PAL genes with *PAL *genes from other plant species, specifically to compare them with isoforms of known biological functions and relevance from other plants. To check for phylogenetic relationships among the different *PAL *genes, 48 gene sequences located in the National Center for Biotechnology Information (NCBI) database and The Institute for Genomic Research (TIGR) annotated database were aligned. Because four of the tomato sequences came from partial cDNAs, a total of 116 nucleotides corresponding to the 3' end of the coding region were considered. Preliminary analysis of the alignment of these sequences revealed extensive homology, therefore this region was used for phylogenetic reconstruction.

The resulting topology grouped *PAL2 *and *PAL6 *within cluster A, which includes most of the isoforms and homologues from other woody dicotyledonous species. One of the genes in this cluster, *PAL1 *from *Populus tremuloides*, is found in non-lignified cells showing accumulation of condensed tannins, while *PAL2 *from the same species, also included in cluster A, appears in lignification structures and conducting elements from the xylem and the phloem; its expression decreases once the lignification process is complete [57]. Osakabe [58] measured levels of four *PAL *genes in *Populus kitakamiensis *stems developing secondary xylem, and found the highest levels for the *palg2b *transcript, which is also in cluster A.

Cluster B includes the other four tomato *PAL *genes (*PAL1*, *PAL3*, *PAL4 *and *PAL5*) and sequences from dicotyledonous plants including *N. tabacum*, *D. carota*, *S. tuberosum*, *C. chinense *and *I. batatas*. *IPBPAL *from *I. batatas *was induced after mechanical damage [59]; *Tag 402 *from *N. tabacum *was induced 4-fold 2 hours after a methyl jasmonate treatment; *AJ539006 *from *N. tabacum *is positively regulated by H_2_O_2 _[60]; expression of *D. carota DcPAL1 *in a cell suspension could be induced by a fungal elicitor, UV-B irradiation or a dilution effect [25].

The clustering of genes from plants like *T. pratense*, *A. thaliana *and the monocot species suggests that *PAL *duplication and divergence has occurred independently within some plant lineages. On the other hand, the presence of genes from tomato and tobacco in different clusters seems to reflect ancient duplication of other *PAL *genes and divergent evolution. Further sequencing of additional *PAL *genes from these and other species may enable more detailed elucidation of the phylogenetic history of the *PAL *gene family.

*PAL2 *and *PAL6 *genes were grouped with genes from other species in which the process of lignification is very active. At the same time, increases in *PAL2 *and *PAL6 *gene expression were only found in inoculated resistant plants. *PAL3*, clustered with those genes from other species involved in the resistance to various stressors, showed increased expression in both inoculated tomato lines. The distribution of the tomato *PAL *genes in two different clusters may reflect functional differences among isoforms, with possible involvement of *PAL6 *and, very probably, *PAL2 *in lignification, and roles for *PAL1*, *PAL3*, *PAL4 *and *PAL5 *in other biological processes.

While some phenolic compounds occur constitutively and serve as pathogen inhibitors in non-host resistance [61,62], others are synthesized *de novo *in response to fungal infection and act as part of an active defense response [63]. In our study, the only detectable change in total phenolic content after *V. dahliae *inoculation was a small but statistically significant increase in total phenolics in roots of inoculated resistant plants at 2 hpi.

To identify differences in the relative amounts of different phenolics, we analyzed the content of bound phenolics by HPLC. We saw a slight increase in ferulic acid levels in roots of inoculated resistant plants at 2 hpi. We also found differences in *p*-coumaric acid, vanillin and *p*-hydroxybenzaldehyde contents at later post-infection times. These phenols are related to cell wall esterification, which enhances plant resistance against fungal enzymes [35]. The most marked changes were observed in the content of *p*-coumaric acid where we found increased levels of this compound in roots of inoculated resistant tomato plants between 16 and 96 hpi. This increase coincided with maximum peroxidase activity in resistant roots, and may be related to the decline seen in the initial increase in H_2_O_2 _at these times points in inoculated resistant plants. *p*-Coumaric acid has a very important role in the maintenance of cell walls in plants. It mediates the cross-linking of lignins to polysaccharides in cell walls of gramineous plants [64]. Vanillin content increased from 96 hpi on, when peroxidase activity was higher in inoculated resistant plants than in control plants of both lines or in inoculated susceptible plants. To summarize, *V. dahliae *infection had a clear influence on phenolic metabolism in the tomato.

Lignification and reinforcement of cell walls are important processes in the response of plants against fungal infection [65-67]. A lignified cell wall is water-resistant and thus less accessible to fungal cell wall-degrading enzymes [68]. Smit and Dubery [69] observed an increase in synthesis and deposition of lignins and similar polymers after exposure of cotton hypocotyls to an elicitor of *V. dahliae*. The active lignification phase was preceded by increased activity of PAL, cinnamyl alcohol dehydrogenase and cell-bound peroxidases. They also found that the response of a resistant cultivar was faster and more intense than that of a susceptible one. Pomar et al. [43] found that inoculation of pepper varieties differing in their degree of resistance against *V. dahliae *triggered a significant increase in the amount of lignin. In our study, inoculation with *V. dahliae *induced a significant increase in the total amount of lignin in tomato roots in both the susceptible line LA3030 and in the resistant line LA3038, although at earlier times in the latter.

Differences in monolignol composition between the lignin of healthy plants and resistant-related lignin in infected plants have been described in several studies of interaction with a variety of pathogens [66,70]. It has also been observed that the monomeric composition and degree of crossing-over in lignins from inoculated pepper plants were closely related to the maintenance of the integrity of the photosynthetic system and thus with tolerance to the presence of the pathogen [43]. From nitrobenzene oxidation, we found that at 16 dpi there was an increase in the proportion of G groups compared to S groups. In addition, at 28 dpi, both cultivars showed an increased proportion of H groups. Presumably, this increase was due to the incorporation of *p*-coumaryl alcohol in the condensed nucleus. However, the possibility that some of the benzaldehyde units quantified by nitrobenzene oxidation could come from hydroxycinnamic acids bound to cell walls, because they share the aromatic skeleton with monolignols, cannot be ruled out.

The ratio between guaiacyl and syringyl moieties has an important influence on the type and frequency of lignin interunit linkages and, consequently, on lignin structure [71]. In our thioacidolysis analyses of tomato lignins, there were remarkable changes in both resistant and susceptible roots. At 16 dpi there was an increase in the G/S ratio in all inoculated plants, consequent to the reduction of S units. This kind of lignification could be analogous to that found in primary walls, where polymerization is rapid and rich in β-5, β-1, β-β, 5-5 y 5-*O*-4 bonds. These young lignins are rich in hydroxyphenyl (H) and guaiacyl (G) groups and poor in syringyl (S) groups. At 28 dpi, the differences between control and inoculated plants disappeared, as a result of a large incorporation of S groups, characteristic of slow polymerization. The chemistry of sinapyl alcohol radicals predicts that their only coupling modes are β-β and β-*O*-4, because the possible resonance structures are R_*O*4 _and R_β _[72]. The β-β mode is less favored than β-*O*-4 at low concentrations, therefore most sinapyl alcohol sources used in lignin biosynthesis are incorporated in polymers rich in β-*O*-4 bonds. Thus, the higher incorporation of syringyl groups observed at 28 dpi in inoculated plants should be accompanied by a higher number of β-*O*-4 bonds and, consequently, by a higher proportion of the linear fraction of lignins.

Another change observed after *V. dahliae *inoculation was the increase in *O*-4 terminal units of DHCA, *p*-coumaric acid, coniferyl alcohol, vanillin and ferulic acid. The increases were greater in the resistant line and were quantitatively highest for ferulic acid. The O-4 terminal unit can act as nucleation points for the growth of lignins after coupling of with a monolignol radical in position β [73]. There was also a remarkable increase in aldehyde groups in inoculated plants, especially in *β-O*-4 coniferyl aldehyde in the resistant line at 28 dpi. The increase observed in the quantity of aldehyde groups in inoculated cell walls was confirmed by FT-IR analysis. The presence of carbonyl groups could have an inhibitory effect on fungal enzymes, because these groups can react with the amino groups of enzymes, inactivating them. In addition, these aldehydes confer a hydrophobic character to lignins, protecting them against the action of cellulolytic enzymes [74].

In summary, our analysis of lignins indicates that there was an increase in lignin synthesis following inoculation with *V. dahliae*. This increase was greater and faster in the resistant line, where two phases were detected. Initially, there was an accumulation of lignins with a high degree of crossing-over, apparently rich in G and H groups, particularly in its condensed nucleus. This increase is accompanied by deposition of phenolic units that possibly act as initiation points that would allow the growth of highly polymerized linear lignins rich in S units in the slower second phase.

## Conclusions

*Ve*-mediated resistance again *Verticillium *spp. is a complex process that triggers molecular responses at several levels, including H_2_O_2 _accumulation, increased peroxidase activity, differential production of phenylpropanoids, specific regulation of *PAL *genes and differential deposition of lignins. These events are most likely the result of the coordinated activation of different defensive responses, resulting in the production of ROS, the induction of expression of defense genes, the production of antimicrobial compounds and the reinforcement of cell walls. The comparison of sequences from *PAL *genes also seems to reveal the involvement of different PAL isoforms in different biological processes. The generation of specific tomato *PAL *mutants, or the identification by TILLING screening of tomato plants with defective *PAL *alleles, together with an analysis of their resistance against pathogens, their relative phenylpropanoid content and the structure of their cell walls, may elucidate the actual function of the various *PAL *isoforms in lignification or other cellular mechanisms.

## Methods

### Plant material

Seeds from the near-isogenic tomato (*S. lycopersicum *cv Gardener) lines LA3030 and LA3038 were provided by the C.M. Rick Tomato Genetics Resource Center (UC Davis, CA, USA). LA3038 carries the *Ve *gene conferring resistance against *Verticillium *spp. and the I gene for resistance to *Fusarium oxysporum *f. sp. *lycopersici*. The seeds were surface-sterilized by immersion in 10% bleach for 30 minutes and thoroughly rinsed before sowing in sterile perlite. One-week-old plantlets were transplanted into individual pots, placed on heating mats and kept in the greenhouse through September and October with a 16:8 h photoperiod at 342 μmol m^-2^s^-1 ^at temperatures ranging from 18 to 25°C. The plants were supplemented once a week with a water soluble fertilizer (N-P-K: 15-2.2-9 ).

### Fungal material

The virulent *V. dahliae *Kleb. isolate VD53 was used [75]. To ensure virulence, the pathogen was freshly isolated from infected plants before each inoculation. After isolation it was cultured on potato dextrose agar (PDA) plates.

### Fungal inoculation

Four-week-old plants were inoculated with inoculum prepared from the *V. dahliae *cultures grown on PDA plates. After 25 days of culture at 25°C in the dark, 5 ml of sterile distilled water was added to each plate and the mycelia were brushed away with a rubber spatula. The suspension was filtered through a double layer of cheesecloth. The conidia were counted in a Thoma chamber and the concentration adjusted to 10^7 ^conidia ml^-1^. One ml of the suspension was directly pipetted onto the soil surface of each pot. Plants in the control group received 1 ml of sterile water. After inoculation, all plants were kept in the greenhouse conditions described above.

### Measurement of H_2_O_2_

Root samples were homogenized in extraction buffer (Tris-acetate 50 mM, pH 5.0) using a mortar and pestle. The mixture was filtered through a double layer of cheesecloth and centrifuged at 14,000 *g *for 30 minutes at 4°C. The supernatant was transferred into a clean tube and the pellet was discarded.

H_2_O_2 _was quantified in the roots of control and inoculated LA3030 and LA3038 plants using the xylenol orange method [76] that is based on the oxidation of Fe^2+ ^ions by peroxide, followed by colorimetric detection of the reaction of Fe^3+ ^with the sodium salt of xylenol orange. Five hundred μl of the reaction mixture (500 μM ferrous ammonium sulfate, 50 mM H_2_SO_4_, 200 μM xylenol orange and 200 mM sorbitol) was added to 500 μl of root crude extract. After 45 minutes, absorbance by the Fe^3+^-xylenol orange complex was measured at 560 nm. Data were normalized with reference to fresh weight and are presented as H_2_O_2 _concentrations (μM).

### Measurement of peroxidase activity

Sample extraction was performed as described above. Peroxidase activity was determined at 25°C in 50 mM Tris-acetate at pH 5.0 and 0.5 mM H_2_O_2_, supplemented with 1 mM 4-methoxy-α-naphthol as the electron donor [43].

### Measurement of phenylalanine ammonia lyase activity

Total PAL enzyme was extracted by the method of El Ghaouth et al. [77]. The root samples were homogenized at 4°C in 50 mM sodium acetate, pH 5.0; the lysate was then centrifuged at 10,000 *g *for 15 minutes and the supernatant was collected. PAL activity was measured in this fraction using the method of Beaudoin-Eagan and Thorpe [78]. The extract was incubated for 2 h at 37°C in 10 mM L-phenylalanine, 0.5 M Tris-HCl, pH 8.0. The reaction was stopped by adding 5 M HCl. The mixture was centrifuged and the amount of *trans*-cinnamic acid formed in the supernatant was measured spectrophotometrically at 290 nm. PAL activity was expressed as μg of cinnamic acid formed per μg of protein.

To confirm PAL activity, an inhibition assay using different amounts of the cinnamic acid derivatives ferulic acid, *trans*-cinnamic acid, coumaric acid and caffeic acid was performed. Total inhibition of commercial PAL (Sigma-Aldrich, Madrid) was found at concentrations of 10^-10 ^μg μl^-1^.

### RNA extraction and cDNA synthesis

Samples from roots, hypocotyls, epicotyls, cotyledons, leaves and flowers were taken from 6-week-old LA3030 and LA3038 plants. Samples from LA3030 and LA3038 roots were harvested at different times after fungal induction and stored at -80°C for further use. Total RNA was extracted from frozen samples using the Aurum™ Total RNA Mini Kit (Bio-Rad, Barcelona) following the manufacturer's instructions. RNA quantity was measured spectrophotometrically and its integrity was confirmed using 1.2% agarose-formaldehyde gel electrophoresis [79]. First-strand cDNA was synthesized from 100 ng of total RNA using the iScript cDNA Synthesis Kit (Bio-Rad), following the protocol supplied by the manufacturer.

### Primer design, PCR and real-time PCR

Sequences for the different tomato PAL genes were retrieved from the databases of the National Center for Biotechnology Information (NCBI) http://www.ncbi.nlm.nih.gov/ (*PAL1 *and *PAL5*) and The Institute for Genomic Research (TIGR) http://www.tigr.org/tdb/agi/ (*PAL2*, *PAL3*, *PAL4 *and *PAL6*). Specific primers were designed using the program Primer-3 [80]; primer sequences are detailed in Table 7. The amplification conditions of the different *PAL *genes were optimized. The thermal cycling conditions consisted of an initial denaturation step at 95°C for 2 minutes followed by 30 cycles at 95°C for 30 s, 60°C for 25 s, 72°C for 50 s, and a final step at 72°C for 5 minutes. The respective PCR products were sequenced and corresponded to the expected amplicons. PCR products were separated on 1% agarose gels and visualized after staining with ethidium bromide.

**Table 7:** Sequence of PCR primers used for quantification of different *S. lycopersicum PAL *genes using RT-PCR.

Primer name	Sequence (5'-3')	Target	Accession number	Fragment length (bp)
**LE02F**	AGTGGCAACCCTTTAATTCG	*S. lycopersicum; PAL1*	TC153702	479
**LE02R**	CATGTCATCATGTTCACAAAGC		M83314	
				
**LE415F**	TGAAGGAATGGAATGGTGCT	*S. lycopersicum*; *PAL2*	TC165415	303
**LE415R**	TGAAAGAAGCCACAAAAGTTCA			
				
**LE86F**	CAGAATTAAAGGCCGTGTTG	*S. lycopersicum; PAL3*	TC153686	295
**LE86R**	TTTCTGGCAAGCATCTAGCA			
				
**LE99F**	CGGTGAGGAGATTGACAAGG	*S. lycopersicum; PAL4*	TC153699	199
**LE99R**	CCTGTAAAGTTGTAGAAATTGAATGAA			
				
**LE88F**	GGTTGGTTAGACAAGAAGTTGGA	*S. lycopersicum; PAL5*	TC153688	404
**LE88R**	TGTCGTAGTGGGCGTGATTA		M90692	
				
**LE67F**	TTGCAAACAGGATCAACGAA	*S. lycopersicum; PAL6*	TC165267	220
**LE67R**	TTGCTTCACTTCACTTCTAACAGACTGG			
				
**LEbtubF**	GGGTAAGATGAGCACAAAGGA	*S. lycopersicum; β-tubulin*	TC153831	440
**LEbtubR**	GGCAGAAATTGAACAAACCAA			

Real-time RT-PCR was performed in 50 μl of a reaction mixture composed of 2.5 μl cDNA, 1X iQ SYBR Green Supermix (Bio-Rad) and 0.3 μM of each gene-specific primer, using an iCycler iQ system (Bio-Rad) and the same thermal cycling conditions described above. The Optical System Software 3.0 (Bio-Rad) was used to analyze the results. RT-PCR specificity was confirmed by identification of a single peak in the melting curve analysis.

The β-tubulin gene was used as a constitutively expressed endogenous control. To determine the amplification efficiencies for the *PAL *and tubulin genes, we used five-fold serial dilutions of cDNA. Efficiencies greater than 95% were obtained in all cases. For quantification, an efficiency-corrected *C_t _*model was used [81]. For the direct comparison of levels of expression among *PAL *genes in roots, the expression of each gene was related to that of the gene with the lowest *C_t _*(*PAL2*). Each test was repeated twice and each measurement was performed in duplicate.

### Phylogenetic analyses

All sequences representing *PAL *genes from different organisms were extracted from the National Center for Biotechnology Information (NCBI) and The Institute for Genomic Research (TIGR) annotated databases. We included 48 nucleotide sequences, restricted to a homologous region of 116 nucleotides from the 3' end of the coding region.

Multiple alignments of the nucleotide sequences were conducted using the BioEdit [82] and ClustalX [83] programs with default parameters as specified by each program. The trees were produced using maximum parsimony with the MEGA program, version 3.1 [84]. Reliability of the resulting topologies was tested by bootstrap (1,000 replicates) for each interior branch of the trees.

### Analysis of phenolic compounds

Root samples were lyophilized and ground in liquid nitrogen, after which 100-300 mg was homogenized in 70% methanol and incubated for 30 minutes at 80°C. After cooling to room temperature, water was added to a volume of 2 ml and samples were centrifuged at 1,300 *g *for 5 minutes. The pellet was then resuspended in 2 ml 70% methanol and centrifuged again using the same conditions. The supernatants from both extractions were combined and extracted twice with ethyl acetate after methanol evaporation under vacuum. The resulting supernatants were evaporated and resuspended in 1 ml methanol. This supernatant was retained as the soluble phenols fraction.

Two ml of 4 N NaOH was added to the pellet from the soluble phenols extraction, saturated with nitrogen, and incubated at 170°C for 2 hours. After cooling, 2 ml H_2_O and 1 ml 35% HCl were added. The reaction mixture was centrifuged for 10 minutes at 3,200 *g *and the supernatant was retained. Phenolic compounds were extracted 3 times with ethyl acetate, then anhydrous sodium sulfate was added and the samples were evaporated to dryness in a Rotavapor R-205 (Buchi, Postfach, Switzerland) and resuspended in 1 ml methanol; this fraction contained linked phenols.

The quantitative determination of free and linked phenolics was accomplished using Folin-Ciocalteu reagent [85], with ferulic acid as the standard. The sum of both fractions was considered to be the total phenols content.

For identification and quantification of individual compounds, the linked phenol samples were analyzed using reverse-phase HPLC on an Alliance system (Waters, Barcelona) equipped with a Waters 996 photodiode detector. The reverse-phase employed a Spherisorb ODS2 C18 analytical column (Waters) with a Spherisorb ODS2 C18 precolumn. Ten μl of each sample was injected and run at a flow of 1 ml min-1 at 25°C. Compounds were detected between 225 and 400 nm, and quantification was performed at 290 nm, using the corresponding standards.

### Cell wall isolation and lignin analysis

Cell walls were prepared using a Triton X-100 washing procedure that included as the last steps three washes with ethanol and three washes with diethyl ether [86]. Lignin quantification was performed using acetyl bromide [87]. Alkaline nitrobenzene oxidation of lignifying cell walls and HPLC analyses were performed essentially as described in Pomar *et al. *[88]. Quantification of *p*-hydroxybenzaldehyde, vanillin and syringaldehyde was accomplished at 280 nm, using the corresponding standards. Thioacidolysis, which solubilizes the β-*O*-4 lignin core, and gas chromatography-mass spectrometry (GC-MS) analyses were performed using the Thermo Finnigan Trace GC gas chromatograph, Thermo Finnigan Polaris Q mass spectrometer and DB-XLB, J&W (60 m × 0.25 mm I.D.) column [86]. Mass spectra were recorded at 70 eV. Quantification of chromatographic peaks utilized total ionic current (TIC) chromatograms. Fourier transform infrared spectra of finely ground cell wall samples were recorded on a Bruker Vector 22 FT-IR spectrophotometer (Bruker Optics Madrid Spain).

### Statistics

All experiments and measurements were performed in triplicate. The Student t-test was used for two-group comparisons and ANOVA followed by an unpaired Student t-test with Bonferroni's correction was used for multiple group comparisons. The differences were considered significant when *P*-value was < 0.05

## Authors' contributions

CG conceived the study, participated in its design, carried out the molecular and biochemical studies, participated in the sequence alignment, performed the statistical analysis and drafted the manuscript. FP designed experiments, analyzed lignin data and drafted the manuscript. ENU participated in the FT-IR experiments. FM participated in the design and coordination of the study. OMdI conceived the molecular components of the study, participated in its design and coordination and helped to draft the manuscript. All authors read and approved the final manuscript.
